# Measuring the depth of invasion in vulvar squamous cell carcinoma: interobserver agreement and pitfalls

**DOI:** 10.1111/his.13883

**Published:** 2019-07-26

**Authors:** Anne‐Floor W Pouwer, Peter Bult, Irene Otte, Michiel van der Brand, Judith van der Horst, Laurette J V Harterink, Koen K van de Vijver, Esther Guerra, Riena P Aliredjo, Steven L Bosch, Johanna M M Grefte, Saskia Zomer, Harry Hollema, Barry de Heus, Saphira Satumalaij, Patricia C Ewing‐Graham, Joanna IntHout, Joanne A de Hullu, Johan Bulten

**Affiliations:** ^1^ Department of Obstetrics and Gynaecology Radboud University Medical Center, Radboud Institute of Health Sciences Nijmegen the Netherlands; ^2^ Department of Pathology Radboud University Medical Center Nijmegen the Netherlands; ^3^ Department of Pathology Rijnstate Hospital Arnhem the Netherlands; ^4^ Dienst Pathologische Anatomie, Laboratorium voor kankerdiagnostiek en pathologie Universitair ziekenhuis Gent Gent Belgium; ^5^ Department of Pathology Hospital Universitari de Bellvitge Barcelona Spain; ^6^ Laboratory for Pathology and Medical Microbiology (Stichting PAMM) Eindhoven the Netherlands; ^7^ Department of Pathology Gelre Hospital Apeldoorn the Netherlands; ^8^ Department of Pathology Canisius‐Wilhelmina Hospital Nijmegen the Netherlands; ^9^ Department of Pathology University of Groningen – University Medical Center Groningen Groningen the Netherlands; ^10^ Department of Pathology Erasmus Medical Center Rotterdam; ^11^ Department of Health Evidence, Radboud University Medical Center, Institute for Health Sciences Nijmegen the Netherlands

**Keywords:** depth of invasion, interobserver agreement, vulvar neoplasm, vulvar squamous cell carcinoma

## Abstract

**Aims:**

The depth of invasion is an important prognostic factor for patients with vulvar squamous cell carcinoma (SCC). The threshold of 1 mm distinguishes between FIGO stages IA and ≥IB disease and guides the need for groin surgery. Therefore, high interobserver agreement is crucial. The conventional and the alternative method are described to measure the depth of invasion. The aims of this study were to assess interobserver agreement for classifying the depth of invasion using both methods and to identify pitfalls.

**Methods and results:**

Fifty slides of vulvar SCC with a depth of invasion approximately 1 mm were selected, digitally scanned and independently assessed by 10 pathologists working in a referral or oncology centre and four pathologists in training. The depth of invasion was measured using both the conventional and alternative method in each slide and categorised into ≤1 and >1 mm. The percentage of agreement and Light’s kappa for multi‐rater agreement were calculated, and 95% confidence intervals were calculated by bootstrapping (1000 runs). The agreement using the conventional method was moderate (κ = 0.57, 95% confidence interval = 0.45–0.68). The percentage of agreement among the participating pathologists using the conventional method was 85.0% versus 89.4% using the alternative method. Six pitfalls were identified: disagreement concerning which invasive nest is deepest, recognition of invasive growth and where it starts, curved surface, carcinoma situated on the edge of the tissue block, ulceration and different measurement methods.

**Conclusions:**

Pathologists reached only moderate agreement in determining the depth of invasion in vulvar SCC, without a notable difference between the two measurement methods.

## Introduction

It is generally accepted that tumour thickness and/or depth of invasion (DOI) is a reliable parameter for predicting the likelihood of regional lymph node involvement and survival in many malignancies, such as cervical, head and neck and colorectal cancers.^1^, ^2^, ^3^ The DOI is also an important prognostic factor in patients with vulvar squamous cell carcinoma (SCC) and determines the need for groin surgery. Early‐stage vulvar SCC is treated by radical local excision of the tumour, with or without inguinofemoral lymph node staging, depending on the DOI.^4^ In patients with a microinvasive carcinoma (DOI ≤1 mm, FIGO stage IA), the risk of inguinofemoral lymph node metastases is negligible and lymph node staging can be safely omitted.^5^, ^6^ In patients with macroinvasive disease (DOI >1 mm, FIGO stage ≥IB), a sentinel node procedure and/or an inguinofemoral lymphadenectomy is indicated. Inguinofemoral lymphadenectomy is also associated with significant morbidity. This morbidity encompasses short‐term morbidity, including wound infection, formation of lymphoceles and/or wound breakdown in up to 85% of the patients, and long‐term morbidity, including lymphoedema, cellulitis and erysipelas in up to 64% of the patients.^7^, ^8^, ^9^ Because of the far‐reaching consequences of inguinofemoral lymphadenectomy, classification of the DOI with a threshold of 1 mm is crucial and high interobserver agreement is important.

**Figure 1:**
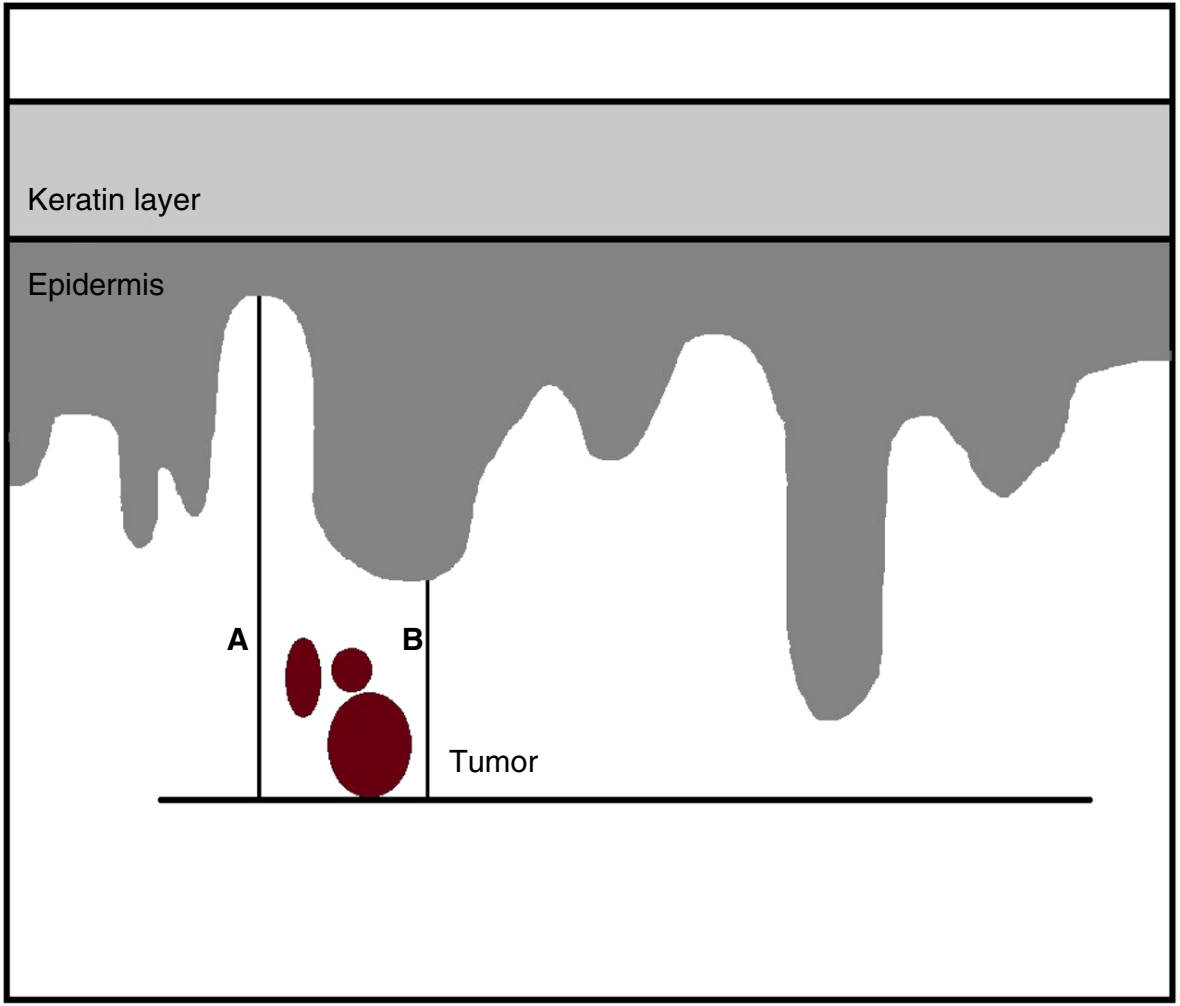
Measurement methods for the depth of invasion in vulvar squamous cell carcinoma. Method A: conventional method; measurement(s) from the epithelial–stromal junction of the most superficial adjacent dermal papillae to the deepest point of invasion. Method B: alternative method; measurement from the most adjacent dysplastic abnormal rete ridge to the deepest point of invasion.

Wilkinson *et al.* 10 have described a number of methods for measuring the DOI in vulvar SCC. The International Federation of Gynecology and Obstetrics (FIGO) recommend to: ‘measure from the epithelial–stromal junction of the most superficial adjacent dermal papillae to the deepest point of invasion’, as shown in Figure 1, method A.^11^ In many carcinomas such as cervical cancer, the depth of invasion is measured from the nearest dysplastic crypt or surface epithelium,^1^ because logically tumour cells will originate from the nearest rete ridges instead of the most superficial dysplastic epithelium. In vulvar cancer, this measurement method (measurement from the most adjacent dysplastic abnormal rete ridge to the deepest point of invasion) is analogous to the method used in cervical cancer (see Figure 1, method B).

This alternative measurement method has been studied by Van den Einden *et al.* 12; they performed a retrospective study comparing the DOI measured by both the conventional and alternative method in a series of vulva carcinoma, and concluded that the alternative method may provide a better reflection of the prognosis. With a cut‐off of 1 mm for both methods, the alternative method resulted in downstaging of the FIGO stage to IA (DOI ≤1 mm) in 9% of the patients (14 of 148). In 13 patients (19%) with FIGO stage IB disease the carcinoma was downstaged to stage IA, in which no groin surgery is indicated. In none of patients was there evidence of lymph node metastasis. However, in one downstaged patient from FIGO stage IIIA isolated tumour cells were present in the lymph node removed by the sentinel node technique.

The question was raised of whether there is a difference in the interobserver agreement when pathologists use the method recommended by FIGO or an alternative method as described above. We aimed to assess the interobserver agreement between pathologists using two different measurement methods and to identify pitfalls in the assessment of the DOI.

## Methods

Slides from biopsies and/or surgical resection specimens of patients treated for vulvar squamous cell carcinoma at the Radboud University Medical Center between 2000 and 2017 were retrieved. An expert gynaecological pathologist (J.B.) reviewed and selected slides for inclusion; both diagnostically challenging and straightforward slides were selected, representing daily practice. In all slides there was a DOI of approximately 1 mm; approximately half the slides showed a DOI ≤1.0 mm and half >1.0 mm at the initial histopathological examination measured by the conventional method. The area of invasion was circled on the slide and all slides were anonymised.

All slides were assessed independently by all participants working in either a gynaecological oncology centre or a referring hospital. The expert pathologist (J.B.) who selected the slides for inclusion did not participate in the study. For each individual slide, participants measured the DOI using both the conventional and the alternative methods using a digital ruler. The digital ruler measures the distance between two locations and a straight line was displayed. Each measurement was reported in mm, with an accuracy of 1 decimal point in an online questionnaire using Castor EDC.^13^ After assessing the slides, the participants recorded how certain they were about each measurement and noted any difficulties and/or comments. Furthermore, the participants were asked what method they used in daily practice and how many years of experience they had.

We based our sample size on a previous study which evaluated the interobserver agreement when assessing the DOI of vulvar SCC.^14^ We estimated that the kappa for interobserver agreement for the DOI ≤1 versus >1 mm using the conventional measuring method would be approximately 0.70 [standard deviation (SD) = 0.10]. With 10 participating pathologists, a power of 80%, an alpha of 5% and a two‐sided 95% confidence interval (CI) of maximal 0.10, 50 slides were required for pathological assessment.^15^ In addition, we included four pathology residents to assess all 50 slides in order to identify differences in the interobserver agreement between residents and pathologists.

The slides were digitally scanned (Pannoramic P250 Flash II; 3DHistech) and distributed to the participants using tEPIS (Trait Enhanced Pathology Image Sharing‐system), a digital pathology platform. The participants were not informed about the original diagnosis, did not receive any clinical information and were not aware of the measurements made by other participants. The participants received Figure 1 as instruction on how to perform both measurement methods. The conventional method was defined as: ‘measurement(s) from the epithelial–stromal junction of the most superficial adjacent dermal papillae to the deepest point of invasion’. The alternative method was defined as: ‘measurement from the most adjacent dysplastic abnormal rete ridge to the deepest point of invasion’.

The annotations made on the slides by each participant were visible to the researcher and were reviewed by the expert gynaecological pathologist (J.B.); this gave the pathologist insight into where exactly the measurement had been made, and allowed review of the discordant slides and analysis of the reasons for discrepancies to identify pitfalls.

### Statistical analysis

For purposes of analysis, the DOI measurements were dichotomised into two categories, DOI ≤1.0 and >1.0 mm, as this categorisation is clinically relevant. The percentage of interobserver agreement was calculated separately for the conventional and the alternative methods for diagnostically challenging and straightforward slides, and for pathologists working in a gynaecological oncology centre or referring hospital. Light’s kappa for multi‐rater agreement was calculated for the conventional method and 95% CIs were calculated by bootstrapping (1000 runs). Kappa values were interpreted as slight (<0.21), fair (0.21–0.40), moderate (0.41–0.60), substantial (0.61–0.80) or almost perfect (0.81–0.99) interobserver agreement.^16^

A slide was arbitrarily defined as discordant if there was agreement on the DOI, classified as microinvasive (DOI ≤1 mm, FIGO stage IA) or macroinvasive (DOI >1 mm, FIGO stage ≥IB) among fewer than seven of 10 pathologists (≤60%), using either the conventional or the alternative method. The statistical software r was used for statistical analysis (version 3.3.2) with the ‘irr’ package.

### Ethics statement

Anonymised residual tissue was used, which was retrieved during regular treatment. According to Dutch law, no specific patient approval is necessary for the use of this material. This study was approved by the local ethical committee (number 2016‐2728) and performed according to the Code for Proper Secondary Use of Human Tissue (Dutch Federation of Biomedical Scientific Societies (http://htpp://federa.org).

## Results

Of the 50 slides selected, 24 (48%) were diagnosed as microinvasive (DOI ≤1 mm, FIGO stage IA) and 26 (52%) macroinvasive (DOI >1 mm, FIGO stage ≥IB) at initial histopathological examination. Ten pathologists assessed all 50 slides; there was a median of 10 years’ experience as a pathologist (range = 0.5–35 years). Five pathologists worked in a gynaecological oncology centre and five in a referring hospital, all within Europe; eight in the Netherlands, one in Belgium and one in Spain. Additionally, four residents, all working in an oncology centre in the Netherlands, assessed all study slides. According to the participating pathologists, microinvasive growth (DOI ≤1 mm, FIGO stage IA) was present in 32–72% and macroinvasive growth (DOI >1 mm, FIGO stage ≥IB) in 22–66% of the study slides using the conventional method; see Table 1. The alternative method resulted in downgrading from macroinvasive growth (DOI >1 mm or FIGO stage ≥IB) into microinvasive growth (DOI ≤1 mm, FIGO stage IA) in 52–80% of the slides assessed as macroinvasive (DOI >1 mm or FIGO stage ≥IB) growth using the conventional method; see Table 1.

**Table 1:** Measurements of pathologists using the conventional method in relation to the original diagnosis and the number of slides downgraded from macroinvasive (DOI >1 mm, FIGO stage ≥IB) to microinvasive (DOI ≤1 mm, FIGO stage IA) using the alternative method to measure the depth of invasion

Pathologist	Microinvasive *n* (%)	Macroinvasive *n* (%)	Not assessed n (%)	Downgraded *n* (%)
Original diagnosis	24 (48)	26 (52)		
1	19 (38)	30 (60)	1 (2)	21/30 (70)
2	21 (42)	29 (58)	0	16/29 (55)
3	19 (38)	31(62)	0	20/31 (65)
4	26 (51)	23 (47)	1 (2)	12/23 (52)
5	20 (40)	30 (60)	0	24/30 (80)
6	17 (34)	33 (66)	0	19/33 (59)
7	36 (72)	11 (22)	3 (6)	6/11 (55)
8	20 (40)	30 (60)	0	23/30 (77)
9	16 (32)	32 (64)	2 (4)	22/32 (69)
10	25 (50)	24 (48)	1 (2)	13/24 (54)

The agreement among pathologists in the assessment of the DOI was moderate (κ = 0.57, 95% CI = 0.45–0.68) using the conventional method. The percentage of agreement among the participating pathologists using the conventional method was 85.0% versus 89.4% using the alternative method. As shown in Table 2, in diagnostically challenging slides the agreement was higher using the alternative compared to the conventional method.

**Table 2:** Agreement among pathologists (*n* = 10) and pathologists in training (*n* = 4) in assessing the depth of invasion

Subgroups	Conventional method (%)	Alternative method
Pathologists		
Overall agreement	85.0	89.4
Slides		
Straightforward (*n* = 30)	86.3	91.3
Diagnostically challenging (*n* = 20)	83.0	86.5
Type of centre		
Oncology (*n* = 5)	88.0	91.6
Referring (*n* = 5)	83.2	88.8
Slides with full agreement	34.0	54.0
Discordant slides (agreement ≥60%)	10.0	8.0
Residents		
Overall	93.5	89.5
Slides		
Straightforward (*n* = 30)	95.8	90.8
Diagnostically challenging (*n* = 20)	90.0	87.5
Slides with full agreement	84.0	72.0
Discordant slides (agreement ≤60%)	6.0	10.0

Pathologists working in an oncology centre reached higher agreement than those from the referring centres for both the conventional method (88.0% versus 83.2%, respectively) and the alternative method (91.6% versus 88.8%, respectively); see Table 2. Using the conventional method, full agreement by the pathologists was obtained in 34% (17 of 50) of the slides and five slides (five of 50, 10%) were considered as discordant; in one slide agreement was 40%, in two 50% and in two 60%. For measurements made by the alternative method, full agreement by the pathologists was obtained in 54% (27 of 50) of the slides and four slides were considered as discordant; agreement was 50% in one and 60% in the others. One slide was included in both groups.

As shown in Table 2, agreement between residents was 93.5% using the conventional method and 89.5% using the alternative method. There was full agreement between all four residents in 84% (42 of 50) and 72% (36 of 50) of the slides, respectively. There were more discordant slides using the alternative method (10%) compared to the conventional method (6%).

Of the 10 participating pathologists, seven (70%) used the conventional method and two (20%) the alternative method to measure the DOI in daily practice. One (10%) pathologist used a combination of the two methods, using the alternative method in tumours with early stromal invasions or microinvasion.

Three of the four (75%) residents used the conventional method in daily practice. One (25%) used a combination of the conventional and alternative methods (the alternative method in certain cases with microinvasion).

All pathologists scored ease of use on a scale from 1 to 5 (1 = very difficult, 5 = very easy). The ease of use for the conventional method was scored as a median 4 of 5 points (range = 1–5), and the alternative method as a median 4 of 5 points (range = 1–4). Half the pathologists (five of 10) scored both methods equally, three pathologists gave the conventional method a higher score and two scored the alternative method more highly.

All pathologists scored how sure they were about their measurement on a scale of 1 to 5 (1 = not sure at all, 5 = very sure). Eight pathologists were equally sure about their measurement using both methods; one pathologist was more sure about the measurements using the conventional method and one using the alternative method. The overall score was median 3 for the conventional method versus median 3 for the alternative method.

**Figure 2:**
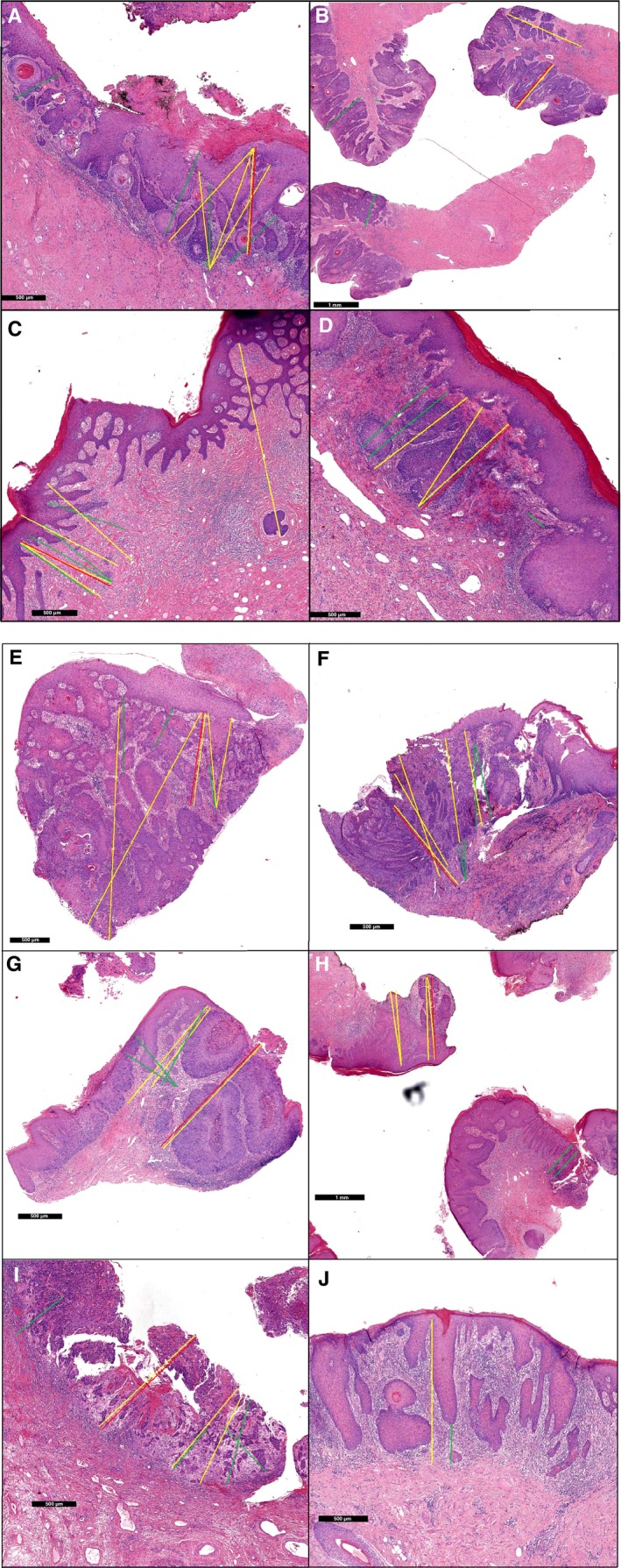
Depth of invasion measured by different pathologists in discordant slides. The yellow and green lines are a measurement of at least one pathologist; macroinvasive [depth of invasion (DOI) >1 mm, FIGO stage ≥IB] measurements are displayed in yellow, microinvasive (DOI ≤1 mm, FIGO stage IA) measurements are displayed in green. The recommended measurement is displayed in red. **A**,**B**,**C**,**G**,**H**, the conventional method was used; **D**,**E**,**F**,**I**, the alternative method was used to measure the depth of invasion. **J**, Both the conventional and alternative method are displayed.

Discordant slides were reviewed by the expert gynaecological pathologist (J.B.) to analyse the reasons for discrepancies. This resulted in the identification of six pitfalls in the assessment of the DOI: (1) disagreement on which invasive nest is deepest (Figure 2A–C), (2) the recognition of whether or not there is, in fact, invasive growth and where it starts (Figure 2B–F), (3) a curved surface (Figure 2G), (4) a carcinoma situated on the edge of the tissue block (Figure 2H), (5) ulceration (Figure 2I) and (6) different methods are used to measure the DOI (Figure 2J). Subsequently, the recommended measurements by the expert gynaecological pathologist are displayed in red in Figure 2.

## Discussion

A threshold of 1 mm in the DOI distinguishes between micro‐ and macroinvasive growth (FIGO stages IA and ≥IB) and guides the need for groin surgery. Reproducibility of this measurement is important and clinically relevant. However, among pathologists there is only moderate agreement (κ = 0.57, 95% CI = 0.45–0.68) in the assessment of the DOI using the conventional method recommended by the FIGO.

The results of our study, showing moderate (κ = 0.51) interobserver agreement between 11 pathologists for classifying the DOI, are in line with another study.^14^ Our study encouraged the participants to use the conventional method to measure the DOI, but only one of 11 participants was able to use this method in all 45 cases. This underlines the difficulty of measuring the DOI in vulvar SCC and the variation in methods of measurement used by pathologists. Our study confirms the result that measuring invasion depth is indeed difficult in vulvar SCC. Additionally, we offer a unique insight into the difficulties of measuring the depth of invasion by the use of digital pathology. In‐depth analyses of all discrepant slides identified six pitfalls. Based on the depicted pitfalls, we formulated recommendations for assessing the DOI in vulvar SCC, as displayed in Table 3. Besides these recommendations, further improvement can be achieved by education, for which the discordant slides and the formulated pitfalls and recommendations of our study are an excellent base.

**Table 3:** Recommendations based on the pitfalls in the assessment of the depth of invasion in vulvar squamous cell carcinoma vulvar squamous cell carcinoma

Pitfalls		Recommendations	Examples, see Figure 2
1.	Recognition which invasive nest is deepest	In tumours ≤1 cm; totally embed the carcinoma If still uncertain, cut at least two deeper levels on the block	A–C
2.	Recognition whether or not there is in fact invasive growth and where it starts	See recommendations of pitfall 1 Tumours >1 cm; enclose one tissue block for every 0.5 cm of the carcinoma	B–F
3.	Curved surface with two or more possible locations of the surface	Measure from the surface resulting in the least favourable depth of invasion	G
4.	Carcinoma situated on the edge of the tissue block	Locate the carcinoma in the middle of the block if possible	H
5.	Ulceration	Sample the carcinoma without ulceration. If not possible, measure from the floor of the tumour ulcer	I
6.	Different measurement methods are used	Use the conventional method. Do not routinely use the alternative method until validated, but if used, state the method of measurement used in the pathology report	J

In case of doubt, if micro‐ or macroinvasive growth (FIGO stages IA or ≥IB) is present in the carcinoma after following the above recommendations, we advise consultation with an expert gynaecopathologist.

We showed that pathologists reach similar agreement for the classification of the DOI into a micro‐ or macroinvasive carcinoma (FIGO stages IA and ≥IB) using the conventional and alternative methods. In contrast, pathologists in training reached higher agreement using the conventional method. This might be explained by recent training concerning the conventional method, and therefore more homogeneity.

The strengths of our study are the international participation in the study, the participation of pathologists working in both referring and oncology centres and the inclusion of slides representing daily clinical practice, i.e. both straightforward and diagnostically challenging slides. In addition, for several reasons, a unique strength is the use of digital pathology. First, digital pathology uses a digital ruler and makes it easier for the pathologist to perform the measurements. Secondly, digital pathology makes it easy to share pictures of the slides between different pathologists for revision and, more importantly, the point of deepest invasion and the measurement made by the pathologist are visible for other consulted pathologists in case of doubt. Thirdly, digital images enabled the researchers to perform in‐depth analyses of the measurements made and allowed analyses of discrepant slides and identification of pitfalls.

A possible limitation of our study is the statistical method used. We dichotomised the measurement of the individual pathologists into ≤1 and >1 mm, as these outcomes are clinically relevant. This may have introduced imprecision, as in some slides the DOI was very close to 1 mm. We were not able to compare the kappa for both methods, because the kappa is dependent on the distribution of the micro‐ and macroinvasive slides between the groups (DOI ≤1 and >1 mm, FIGO stages IA and ≥IB). This distribution was different for both methods, as the alternative method is more likely to result in more carcinomas being classified as microinvasive (DOI ≤1 mm, FIGO stage IA) compared to the conventional method. Another limitation is the selection of slides with a DOI of approximately 1 mm instead of consecutive series. This might have resulted in an underestimation of the interobserver agreement, and an overestimation of the percentage of slides downgraded from stage ≥IB, measured by the conservative method to FIGO stage IA measured by the alternative method.

In conclusion, this study showed only moderate agreement between pathologists classifying the DOI into micro‐ and macroinvasive vulvar SCC (FIGO stages IA and ≥IB) using the conventional measurement method recommended by the FIGO, and similar agreement using the alternative method. This study showed that the alternative method is suitable for pathologists to measure and classify the DOI in vulvar SCC. However, before implementing this method in daily clinical practice, future research should be performed to determine if the alternative method leads to a better reflection of the prognosis and of whether a new threshold needs to be defined to reflect biological tumour behaviour.

## Conflict of Interests

None to declare.
